# Comparative evaluation of orthodontic mini-implants hygiene protocols on subgingival bacterial load

**DOI:** 10.1590/2177-6709.30.1.e2524108.oar

**Published:** 2025-03-24

**Authors:** Amanda Osório Ayres de FREITAS, Ana Paula Vieira COLOMBO, Celuta Sales ALVIANO, Daniela Sales ALVIANO, Renata Martins do SOUTO, Deborah Catharine de Assis LEITE, Isabela Lopes Vale Pedrosa LIMA, Matilde da Cunha Gonçalves NOJIMA

**Affiliations:** 1 Federal University of Rio de Janeiro, Dental School, Department of Pediatric Dentistry and Orthodontics (Rio de Janeiro/RJ, Brazil).; 2 Federal University of Rio de Janeiro, Center for Health Sciences, Department of Oral Microbiology (Rio de Janeiro/RJ, Brazil).; 3 Federal University of Rio de Janeiro, Center for Health Sciences, Department of Surface Structure of Microorganisms (Rio de Janeiro/RJ, Brazil).; 4 Federal Technological University of Paraná, Postgraduate Program in Computational Technologies for Agribusiness - PPGTCA (Medianeira/PR, Brazil). Federal Technological University of Paraná, Postgraduate Program in Biotechnology PPGBIOTEC (Dois Vizinhos/PR, Brazil).; Postgraduate Program in Biotechnology PPGBIOTEC, Federal Technological University of Paraná, Dois Vizinhos, PR, Brazil

**Keywords:** Orthodontics, Oral hygiene, Oral bacteria, Real-time PCR, Ortodontia, Higiene bucal, Bactérias bucais, PCR em tempo real

## Abstract

**Objective:**

The aim of this study was to evaluate the effectiveness of four hygiene protocols for orthodontic mini-implants in reducing the subgingival bacterial load in the peri-implant sulcus.

**Methods:**

Thirty-nine healthy individuals who had fifty-nine as-received mini-implants (20 men, 19 women, 20 to 42 years old) were randomly distributed into four groups of hygiene protocols: mechanical hygiene (M); mechanical hygiene associated with 0.12% digluconate chlorhexidine (CHX), 0.03% triclosan (T), or 0.05% cetylpyridinium chloride (CP). All individuals were instructed regarding the hygiene procedures (T0). For bacterial load analysis, the gingival crevicular fluid from peri-implant sulcus was collected and submitted to quantitative real-time PCR at baseline (T1) and after 21 days following the hygiene protocols (T2). Wilcoxon test was applied for intergroup comparisons, whereas differences among groups at each time point were examined by Kruskal-Wallis test. The significance level was 5%.

**Results:**

Significant difference was detected between baseline and post-protocol times for bacterial total counts, comparing intergroup results, except for mechanical hygiene associated with cetylpyridinium chloride (M p=0.018, CHX p=0.028, T p=0.012, CP p=0.065). No significant difference was detected among the evaluated methods (p=0.181).

**Conclusions:**

The mechanical hygiene of orthodontic mini-implants itself was capable to reduce total bacteria load and keep devices clean. Commonly, orthodontists prescribe, in addition to mechanical biofilm removal, some protocols combining adjunctive chemical agents as chlorhexidine. The authors believe that results have large importance for dental community, as they can protect patients from overtreatment.

## RESUMO

**Objetivo:**

O objetivo do presente estudo foi avaliar a eficácia de quatro protocolos de higienização na redução da carga bacteriana subgengival no sulco peri-implantar de mini-implantes ortodônticos.

**Métodos:**

Trinta e nove indivíduos saudáveis com cinquenta e nove mini-implantes instalados conforme recebidos (20 homens, 19 mulheres, de 20 a 42 anos) foram distribuídos aleatoriamente em quatro grupos de protocolos de higienização: higienização mecânica (M); higienização mecânica associada ao digluconato de clorexidina (CHX) 0,12%, triclosan (T) 0,03% ou cloreto de cetilpiridínio (CP) 0,05%. Todos os indivíduos foram orientados quanto aos procedimentos de higienização (T0). Para análise da carga bacteriana, o fluido crevicular do sulco peri-implantar foi coletado e submetido à PCR quantitativa em tempo real no início do estudo (T1) e após 21 dias de utilização dos protocolos de higienização (T2). O teste de Wilcoxon foi aplicado para comparações dentro dos grupos, enquanto as diferenças entre os grupos, em cada tempo de avaliação, foram investigadas pelo teste de Kruskal-Wallis. O nível de significância foi de 5%.

**Resultados:**

Foi detectada diferença significativa entre os tempos inicial e pós-protocolo nas contagens bacterianas totais, comparando os resultados intergrupos, exceto para higienização mecânica associada ao cloreto de cetilpiridínio (M p=0,018, CHX p=0,028, T p=0,012, CP p=0,065). Não foi detectada diferença significativa entre os métodos avaliados (p=0,181).

**Conclusões:**

A própria higienização mecânica dos mini-implantes ortodônticos foi capaz de reduzir a carga bacteriana total e manter os dispositivos limpos. Comumente, os ortodontistas prescrevem, além da remoção mecânica do biofilme, alguns protocolos que combinam agentes químicos coadjuvantes como a clorexidina. Os autores acreditam que os resultados têm grande importância para a comunidade odontológica, uma vez que poderiam evitar o sobretratamento dos pacientes.

## INTRODUCTION

Control of skeletal orthodontic anchorage depends mainly on the stability of the temporary anchorage device (TAD), which can be influenced by the skill of the operator during surgical procedure, quality of the host bone, anatomical region selected for TAD insertion^1^ ^-^ ^4^, magnitude of the orthodontic force applied, the presence of trauma, and biofilm control around the device.^1^ ^,^ ^3^ ^-^ ^5^ ^,^ ^6^

Adequate dental biofilm control around mini-implants (MI) is a key condition for the prolonged maintenance of these devices, since poor hygiene can lead to peri-implant inflammation and loss of device stability.^1^ ^,^ ^5^ ^-^ ^14^

Immediately after being exposed to the intraoral environment, the surface of the MI is covered by the salivary acquired film, promoting the adhesion of early colonizers. These bacteria create favorable conditions for subsequent adhesion of late colonizers, which comprise many periodontal pathobionts frequently associated with inflammation and periodontal diseases.^9^ ^,^ ^15^ ^-^ ^17^

**Figure 1:**
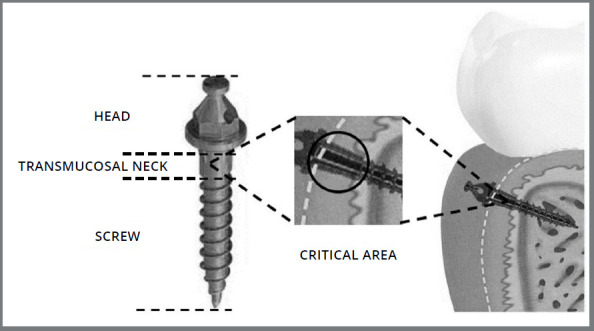
Orthodontic mini-implant and its transmucosal neck, evidencing the critical area.

The sulcus formed between the transmucosal neck of mini-implants and the gingiva keeps the surface of the MI in close contact with the oral mucosa, creating a critical area for dental biofilm accumulation (Fig 1). After installation of the MI, failures in the hygiene around the peri-implant gingival sulcus result in biofilm accumulation, with posterior peri-implant inflammation and loss of its natural seal, compromising the permanence of the MI in the oral cavity.^5^ ^,^ ^7^ ^,^ ^9^ ^-^ ^14^ ^,^ ^17^ ^,^ ^18^

In addition to mechanical biofilm removal, some protocols combining adjunctive chemical agents have been proposed to improve biofilm control on the MI.^5^ ^-^ ^7^ ^,^ ^10^ ^,^ ^12^ ^,^ ^16^ Despite the importance of reducing factors that favor microbial adhesion and biofilm formation, there is no consensus about any particular efficient method for microbial control around mini-implants. Therefore, the aim of the present study was to evaluate the efficacy of four hygiene protocols for microbial control of orthodontic MIs in reducing the total number of subgingival biofilm bacteria. 

## MATERIAL AND METHODS

### SAMPLE POPULATION: ELIGIBILITY CRITERIA AND ETHICAL ASPECTS

**Figure 2:**
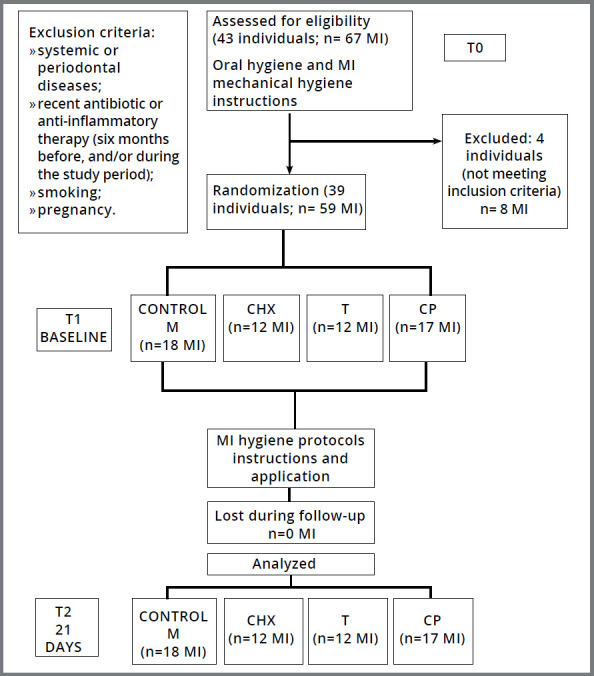
Flowchart for the clinical trial.

As depicted in Figure 2, 39 individuals (20 men and 19 women, 20 to 42 years old) attending the Clinic of Orthodontics at Federal University of Rio de Janeiro (UFRJ, Brazil) were selected for the present study. The exclusion criteria were: presence of any systemic or periodontal diseases, use of antibiotics or anti-inflammatory drugs six months prior to or during the study, smoking and pregnancy. All participants signed a consent form previously approved by the Research Ethics Committee of the Institute of Collective Health Studies at UFRJ (protocol #50/2011; report #149/2011). Fifty-nine MIs (SIN™, São Paulo/SP, Brazil) with 1.4 mm in diameter, 1.0 mm in transmucosal neck and 8.0 mm long were installed at the attached gingiva in the posterior region of maxilla and mandible, varying in number from 1 to 4 MIs per participant, with a range of 30 to 180 days since the installation of the MI. Participants received written general oral hygiene instructions, were trained by a professional and oriented to follow the instructions during 30 days, in order to standardize the hygiene procedures and oral hygiene conditions at T0 (before baseline) (Fig 2).

### RANDOMIZATION AND HYGIENE PROTOCOLS

Patients were enrolled during the orthodontic appointment, when they were assigned to consecutive and increasing numbers. A computer-generated table of random numbers was used for simple randomization of participants. Then, patients were distributed into the following four groups, according to the MI hygiene methods (Fig. 2): mechanical hygiene (M) (interdental brush Colgate^®^, São Paulo, Brazil and toothpaste Colgate^®^ Tripla Ação, São Paulo, Brazil), mechanical hygiene associated with 0.12% chlorhexidine (CHX) (Colgate^®^ PerioGard, São Paulo, Brazil), mechanical hygiene associated with 0.03% triclosan (T) (Colgate^®^ Plax Classic, São Paulo, Brazil), or mechanical hygiene associated with 0.05% cetylpyridinium chloride (CP) (Cepacol^®^ Menta, São Paulo, Brazil). To standardize the hygiene methods, all individuals received written oral hygiene instructions and were trained by a professional. All oral hygiene procedures were performed three times a day, after meals, using toothbrush, dental floss, interdental brush, dentifrice and, when applicable, the chemical agent. The interdental brush and the dentifrice were also used for mechanical hygiene of mini-implants and, according to the experimental group, the interdental brush was moistened with the respective chemical agent and applied around the MI, after the mechanical hygiene. The toothbrush (Twister Colgate^®^, São Paulo, Brazil), the dental floss (Colgate^®^, São Paulo, Brazil), the dentifrice, the interdental brush and the chemical agents, over-the-counter commercial mouthwashes, were provided to the participants for the entire period of the study protocol. 

## MICROBIOLOGICAL ANALYSIS

### SAMPLE COLLECTION

**Figure 3:**
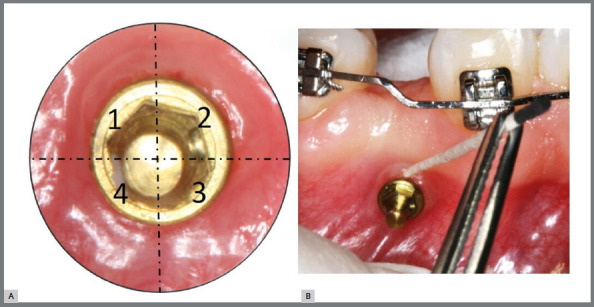
A) Four subgingival sites selected for gingival crevicular fluid collection. B) Sampling from mini-implant sulcus with #40 endodontic paper points.

The gingival crevicular fluid (GCF) in the sulcus formed between the transmucosal neck and the attached gingiva was collected at baseline (T1) and 21 days after application of the hygiene protocols (Post-protocol/ T2).^5^ Prior to sampling, supragingival biofilm was removed with sterile gauze and the area around dental surface and MI’s head was dried with compressed air. The anchorage devices were isolated with sterile cotton rolls, to avoid potential contamination with saliva.^19^ Four endodontic paper points #40 (Endo Points^TM^, Manacapuru/AM, Brazil) were gently inserted in four different areas at MI gingival sulcus, between the MI’s transmucosal neck and the attached gingiva. The paper points were kept inside the sulcus for 45 seconds to absorb the GCF (Fig 3).^19^ Then, they were placed into sterile microtubes containing 500 µL of Tris EDTA buffer and maintained at -20°C until laboratory process.

### DNA EXTRACTION

Genomic DNA was obtained from the GCF samples by a proteinase K method.^15^ After thawing, tubes were vortexed for 60 seconds and the paper points, removed. The suspension was centrifuged; the supernatant, discarded and the pellet, suspended into a 50 µL buffer containing 44 µL of TE, 5 µL of Tween 20.5%, and 1 µL of Proteinase K 10 mg/mL. The total genomic DNA concentration of each sample was measured (NanoDrop^TM^ 2000, Thermo Scientific, Waltham, MA, USA) and adjusted to provide ideal conditions for further amplification.

### REAL-TIME PCR QUANTITATIVE ANALYSIS

Bacterial quantitative analysis was performed using real-time PCR targeting 16S ribosomal RNA. A total of 354GCF samples from baseline and 21 days after hygiene protocols were processed. Power SYBER Green PCR Master Mix (Applied Biosystems, Foster City, CA, USA) was used, with in a total reaction volume of 20 µL. The universal primers selected were 5’ - GAT TAG ATA CCC TGG TAG TCC AC - 3’ and 5’ - TAC CTT GTT ACG ACT T - 3’.^20^ The amplification reaction comprised 5 µL of genomic DNA (3 ng/µL), 10 µL of MIX, 0.4 µL of each primer, 0.4 µL of ROX (1:10), to control variation in volume and evaporation along the reaction, and 3.8 µL of ultrapure water. The mix reactions were distributed into 96-well culture plates, sealed, centrifuged and inserted into the thermocycler, for amplification. The amplification programmed cycles were: 1 cycle 95ºC/10min; 40 cycles 95ºC/1min, 40 cycles 52ºC/1min and 40 cycles 72ºC/1min. Amplifications were measured at 78ºC. The fluorescence was monitored in every cycle. Standard curves with 1:1 ratio between the number of copies and the number of bacterial cells were used as reference to acquire the results and determine bacterial levels in the analyzed samples.^20^ The curves concentration ranged from 10^2^ to 10 ^7^, based on DNA extracted from *Enterococcus faecalis* ATCC 29212 strain. ^20^ During reaction, the fluorescent signals were revealed in graphics, and numeric values were obtained as results of mathematical calculus processed by the software ABI 7500 v. 2.0.4 (Applied Biosystems, Foster City, CA, USA).

## STATISTICAL ANALYSIS

Data were analyzed using the SPSS 21.0 (Statistical Package for Social Sciences; SPSS Inc., Chicago, IL, USA). Descriptive analysis was presented as mean values and standard deviation of bacterial counts, transformed to log10. Intergroup differences in mean log counts from baseline to post-protocol times were evaluated by the Wilcoxon test, whereas differences among groups at each time point were examined by Kruskal-Wallis test. The significance level was 5%.

## RESULTS

**Figure 4:**
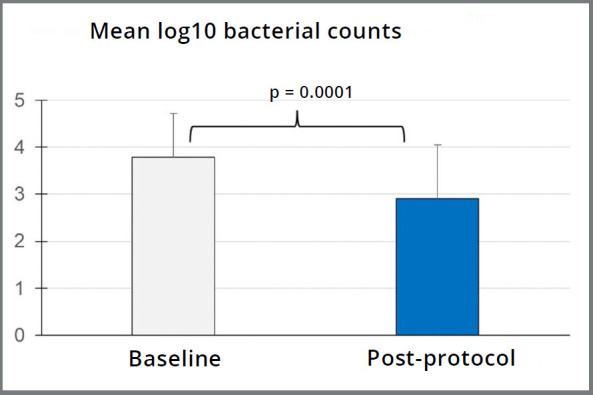
Overall changes in mean counts (log10) of total subgingival bacteria around mini-implants, before and 21 days after introduction of hygiene protocols (Wilcoxon test, p<0.001).

Regardless of the hygiene protocol, an overall significant reduction (approximately 19% mean reduction) in bacterial counts was observed after oral hygiene introduction (p<0.01, Fig 4). Table 1 shows the mean log10 bacterial counts determined by Real time PCR in the GCF samples from the experimental groups, at baseline and 21 days after the hygiene protocols. Except for the CP group, all protocols were able to reduce significantly the subgingival bacterial counts after their application (p<0.05, Wilcoxon test). According to the results presented in Table 1, although the percent of reduction of the subgingival bacterial counts after hygiene protocol administration was greater in the T group (30% reduction), compared to the M (17%), CHX (23.6%) and CP (9%) groups (Table 1), no significant differences among these protocols were observed (p>0.05, Kruskal-Wallis test).

**Table 1:** Intragroup and intergroup comparisons regarding mean changes (SD) in the number of subgingival bacterial cells (log 10) detected at baseline and after hygiene protocols.

Hygiene protocol groups	M	CHX	T	CP	Intragroup p value*
	(n=18)	(n=12)	(n=12)	(n=17)	
Baseline (T1)	3.69 (1.05)	3.82 (0.32)	3.68 (0.96)	3.91 (1.13)	0.988
Post-protocol (T2)	2.76 (1.11)	2.90 (1.03)	2.52 (1.07)	3.32 (1.24)	0.181
Intergroup p value**	0.018	0.028	0.012	0.065	

M = mechanical cleaning; CHX = mechanical cleaning plus use of 0.12% of digluconate of chlorhexidine; T = mechanical cleaning plus use of 0.03% triclosan; CP = mechanical cleaning plus use of 0.05% cetylpyridinium chloride. *Kruskal-Wallis test; ** Wilcoxon test.

## DISCUSSION

The simple presence of orthodontic fixed appliances disturbs oral hygiene, interfering with mechanical removal of dental plaque and favoring the colonization of oral pathogens.^21^ Like other orthodontic devices, the mini-implants are easily colonized, being difficult to keep clean, since the peri-implant area is usually difficult to access.^1^

The present study was not blinded for examiners or for patients. Research participants were unaware of the interventions and the products used by different groups. Therefore, the treatment effect of each hygiene strategy may have been affected by the patient’s motivation when applying the suggested protocol, especially if they were well informed about the product they were using. However, the oral hygiene instructions were reinforced to all patients at each appointment, in order to reduce potential bias in the results. As examiners were trained and calibrated to realize the collection procedures, the authors assumed that the possibility of interference in the results could be reduced, although they might know the hygiene products effects on plaque control. 

Another limiting factor in the present study was the difficulty in recruiting patients since the installation of the mini-implants, so that all evaluated devices would have been exposed to the oral environment and its influences for the same period of time. This difficulty was minimized by the equivalence of oral health status and plaque control of participants, by oral hygiene instruction and training for 30 days before the baseline. 

The period of time that mini-implants remain in the oral cavity is variable. In an attempt to increase the number of devices evaluated, the time interval determined for this study was restricted to 21 days ^5^. The authors intend to extend the evaluation time and to try to associate clinical periodontal parameters in further studies, in order to observe whether the difference between the proposed strategies remains without significant statistical difference, confirming the present results. 

Previous published data reported that brushing the teeth with fluoride dentifrice is enough to prevent peri-implantitis and reduce the quantity of pathogenic species from the oral microbiota.^19^ ^,^ ^22^ Nevertheless, some authors showed that mechanical debridement alone on implant surfaces does not remove all adhering micro-organisms, and should be complemented with other peri-implant plaque control approaches, such as the use of antiseptics.^16^ Therefore, a combination of chemical agents and mechanical therapy should be preferred in order to effectively diminish the levels of periodontal pathogens and oral biofilm formation on titanium surfaces, including orthodontic mini-implants.^10^ ^,^ ^12^ ^,^ ^23^ ^-^ ^25^

The current investigation compared the short-term efficacy of MI hygiene protocols on reducing gingival plaque bacterial load. In order to achieve better local action, the protocols involved the topical use of the mouthwashes around TAD with interdental brush. Overall, the data showed that cleaning the orthodontic MI with interdental brush with or without additional use of topical antiseptics was equally efficient in reducing non-specific total bacterial load on these devices, except for the cetylpyrydinium chloride 0.05% group.

A systematic review showed significant reduction of dental plaque retention around dental implants after the use of cetylpyrydinium chloride at different concentrations, as 0.07% and 0.05%.^26^ Although it is not clear, regarding the lack of 0.05% cetylpyridinium chloride effect on bacterial counts reduction, it is possible that the 0.05% concentration used in the present investigation was not ideal for a topical application, considering that this antimicrobial is known to present low retention on oral surfaces. Specially in orthodontic patients, the cetylpyridinium chloride mouthwash has limited effect in reducing plaque accumulation and periodontal inflammation.^21^

The effectiveness of protocols including use of toothbrushes, dentifrice with triclosan, and other chemical agents is not established in the management of peri-implant mucositis.^27^ Sreenivasan et al^28^ evaluated the clinical and microbiological effects of 0.3% triclosan dentifrice on dental implants after six months and concluded that triclosan dentifrice reduced microbial load, when compared with fluoride dentifrice, preventing peri-implantitis around dental implants. The use of interdental brush with dentifrice and 0.3% triclosan mouthwash was also efficient in reducing total bacterial load around orthodontic mini-implants, according to the present results.

Although 0.12% chlorhexidine is considered an efficient chemical agent for peri-implant inflammation control, after a six months follow-up analysis, the efficacy of 0.12% gluconate chlorhexidine for treatment of peri-implant mucositis on dental implants, associated with periodontal basic therapy, did not show statistical difference, when compared to the control group, treated only with periodontal basic therapy.^29^ The previous published data corroborates the present results, that evidenced bacterial load reduction, but did not show significant difference between the mechanical treatment alone and the association with topical application of 0.12% chlorhexidine. 

Therefore, patient-administered mechanical plaque control alone should be considered the standard of peri-implant mucositis management.^27^ The present findings reinforce the importance and efficiency of mechanical plaque removal as the main hygiene method to maintain healthy oral tissues around orthodontic mini-implants. 

## CONCLUSIONS

The hygiene protocols for plaque control in orthodontic MIs involving mechanical plaque removal using interdental brushes and dentifrices alone or combined with topical 0.12% chlorhexidine or 0.03% triclosan were similarly effective in reducing bacterial load at the gingival sulcus of the MIs. Commonly, orthodontists prescribe, in addition to mechanical biofilm removal, some protocols combining adjunctive chemical agents, as chlorhexidine. The authors believe that the present results have large importance for the dental community, since they can simplify and make the hygiene procedure for cleaning mini-implants less expansive, minimizing the prescription of additional chemical agents for compliant and motivated patients. 
